# Clinical Lecture—Intestinal Obstructions

**Published:** 1846-08

**Authors:** 


*Clinical Lecture—Intestinal Obstructions.* By Professor Chomel.—A patient recently died in our wards (Hotel Dieu) from the presence of intestinal obstruction. She was a woman aged nineteen, of a strong constitution, and enjoying usually good health. Six months before her death she miscarried, and presented afterwards all the symptoms of peritonitis. It was impossible, on inspection of the preparation, to doubt that the inflammatory symptoms originated on the surface of the womb. It is, gentlemen, a remark we have often had an opportunity of making, that no disease is more rare than primary spontaneous peritonitis; when, therefore, you are called to a case of inflammation of the peritoneum, you should first endeavour to ascertain by what disease it has been produced, and in the detection of this primary malady you may find the most urgent practical indications. Our patient had, therefore, been affected with peritonitis consequent upon inflammation of the uterus after miscarriage. These symptoms had subsided, when, about a fortnight before death, new pains appeared in the abdomen, attended with frequent, almost continuous, vomiting. When she was admitted into hospital the extremities were cyanosed, the pulse 120. The vomiting continual, and the peristaltic motion of the intestines visible through the abdominal walls. The belly was not meteoric, and the matter vomited, at first of a bilious nature, soon acquired a fæcal odour, and left no doubt of the presence of an obstruction in the course of the intestines. Various purgatives were unsuccessfully exhibited, and the patient lived only forty-eight hours after admission into our wards. On dissection, the omentum was found to adhere at one point to the inner surface of the pelvis, and marks of inflammation were readily detected all over the serous membrane. In the hypogastric region solid adhesions were discovered; the first seven feet of the small intestine were in a state of considerable dilatation, equalling in size the large intestine. The digestive tube was, on the contrary, atrophied from the union of its two superior with its inferior third, and was much narrower than in health. In the exact spot where the exaggerated dilatation of the intestine ceased, the ileum was strictured by a portion of intestine lying across it, and firmly attached by one of its extremities to the brim of the pelvis, and by the other to the omentum. The occlusion occupied the left side, and above it the small intestine was half twisted on its axis. In several other parts of the pelvis peritoneal adhesions seemed also to interfere more or less with the circulation of matter in the cavity of the digestive organs, the lower parts of which contained neither gas nor stercora ; above the stricture was accumulated a large quantity of liquids. We found also an ulceration of the rectum, which does not seem to have any connection with the causes of death; the womb was healthy.

The disease of which this poor woman presented an example has been but imperfectly studied. The names of ileus, volvulus, meserere, iliac passion, &c. have been generally employed to designate it, In the description of a disease it is not our custom to begin with the anatomical alteration; it appears to us more rational to investigate first the causes, symptoms, progress, and treatment of a disease, before entering upon the examination of the physical changes it has produced ; but, in the description of some maladies, we are forcibly obliged to modify this general rule, because the anatomical alteration is the chief point—the important feature, without a correct knowledge of which no insight into the nature of the disease can possibly be obtained.

*Morbid Anatomy.*—The anatomical cause of obstruction will be found occasionally to reside in the intestinal cavity, in the walls of the digestive tube, or in some neighbouring organs. Thus we find that strangulation may be caused by epiploic adhesions situated in any part of the abdominal cavity. The appendices of the omentum, the appendix vermiformis, occasionally incarcerate a portion of intestine, and we have seen in the melancholy case which we related at the beginning of this lecture, that an intestinal convolution may press like a ring against another part of the tube, and obliterate its cavity. It is not impossible that strangulation of a portion of intestine may take place through a laceration of the diaphragm, and lacerations of the omentum or mesentery have been observed to give passage to and to strangulate a convolution of the ileum or jejunum. Another form of intestinal obstruction, *intussusception*, is not unfrequently observed ; not that intussusception is always followed by fatal symptoms. A portion of the small intestine may accidentally be invaginated in the neighbouring part of the digestive canal without the production of any severe accidents, particularly in children. You will often find in them intussusception to be merely a cadaveric alteration, or to have been produced during the last moments of life. But the most dangerous of all forms of intussusception is undoubtedly the reception of the small into the large intestine. The immobility of the colon, and the presence of the ileo-cœcal valve, render constriction inevitable, and its consequences more dangerous. We should also say that if this is the severest, it is also the least frequent form of invagination. The obstacle once established, fæcal matter cannot pass onwards; the venous circulation of the part is arrested, and symptoms of inflammation appear. The intestine may also be completely twisted round, so as to be completely impervious in one spot. This is an uncommon sort of obstruction; but has been observed in several instances, and in one case in particular, related by M. Andral, the entire mass of the small intestine was twisted by a universal movement of rotation, so as to present an insurmountable obstacle to the progress of its contents.—*Lond. Med. Times.*

